# Apoptosis of Eosinophil Granulocytes

**DOI:** 10.3390/biology9120457

**Published:** 2020-12-10

**Authors:** Martina Zustakova, Lucie Kratochvilova, Petr Slama

**Affiliations:** Department of Animal Morphology, Physiology and Genetics, Faculty of AgriSciences, Mendel University in Brno, Zemedelska 1, 613 00 Brno, Czech Republic; martikzustik@seznam.cz (M.Z.); xkratoc3@node.mendelu.cz (L.K.)

**Keywords:** apoptosis, inflammation, allergy, eosinophil granulocyte

## Abstract

**Simple Summary:**

Eosinophil granulocytes (eosinophils) belong to the family of white blood cells that play important roles in the development of asthma and various types of allergy. Eosinophils are cells with a diameter of 12–17 µm and they originate from myeloid precursors. They were discovered by Paul Ehrlich in 1879 in the process of staining fixed blood smears with aniline dyes. Apoptosis (programmed cell death) is the process by which cells lose their functionality. Therefore, it is very important to study the apoptosis of eosinophils and their survival factors to understand how to develop new drugs based on the modulation of eosinophil apoptosis for the treatment of asthma and allergic diseases.

**Abstract:**

In the past 10 years, the number of people in the Czech Republic with allergies has doubled to over three million. Allergic pollen catarrh, constitutional dermatitis and asthma are the allergic disorders most often diagnosed. Genuine food allergies today affect 6–8% of nursing infants, 3–5% of small children, and 2–4% of adults. These disorders are connected with eosinophil granulocytes and their apoptosis. Eosinophil granulocytes are postmitotic leukocytes containing a number of histotoxic substances that contribute to the initiation and continuation of allergic inflammatory reactions. Eosinophilia results from the disruption of the standard half-life of eosinophils by the expression of mechanisms that block the apoptosis of eosinophils, leading to the development of chronic inflammation. Glucocorticoids are used as a strong acting anti-inflammatory medicine in the treatment of hypereosinophilia. The removal of eosinophils by the mechanism of apoptosis is the effect of this process. This work sums up the contemporary knowledge concerning the apoptosis of eosinophils, its role in the aforementioned disorders, and the indications for the use of glucocorticoids in their related therapies.

## 1. Introduction

Eosinophils play a key role in fighting large multicellular pathogens, such as nematode parasites. Although eosinophils are capable of bactericidal phagocytosis in vitro, it is not possible to effectively prevent bacterial infection in vivo if the function of neutrophils is reduced, such as in the case of pharmaceutically-induced neutropenia or leukocyte adhesion deficiency syndrome [1]. Eosinophils are also able to phagocytose bacteria after recruitment into the lung during allergic lung inflammation [2]. Eosinophils have also been ascribed a prominent protective role in allergic reactions due to the cytotoxic production of mast cells. A unified view of eosinophils as protective cells remained largely unchallenged for many decades and was further supported by the discovery that the proteins in eosinophilic granules are toxic to a variety of parasitic helminth species [3]. Specht et al. demonstrated the functions of eosinophil peroxidase and major basic protein 1 for the elimination of filarial nematodes [4]. It was later discovered, however, that mature eosinophils, particularly the activated ones, are very dynamic and metabolically active cells with a wide variety of functional capabilities that could contribute to a number of important immune defense mechanisms. From this point of view, eosinophils appear to be more similar to macrophages than to neutrophils [3]. It is therefore clear that eosinophils have a significant influence on the potential for tissue injury. This supports the opinion that eosinophils are strong inflammatory cells which, depending on the situation, are just as capable of damaging as they are of protecting their host. For example, hypereosinophilic syndrome is classified as a separate disease related to eosinophilia and tissue injury [5]. There is also considerable evidence of a relationship between the number or activity of eosinophils and the severity of inflammatory diseases such as asthma and chronic obstructive pulmonary disease [6]. Recently obtained data has shown that in patients with COVID-19, eosinopenia developed during the infection. This data suggests that an elevation of the number of eosinophils could indicate an improvement of this disease [7].

## 2. Protective Role of Eosinophils

The main protective role of eosinophils is based on the host defense against parasite invasion. This is an acknowledged fact even though it is based primarily on in vitro studies. The relationship between eosinophils, the immune system, and parasitic helminths is nevertheless very complicated. A number of studies have demonstrated the chemoattractant activity of eosinophils in relation to many factors in vitro, including products from attacking helminths. It was previously found that products of lung fluke *Paragonimus westermani* causing pulmonary or extrapulmonary paragonimiasis induce eosinophil apoptosis [8].

The unique task of interleukin (IL)-5 in producing, activating, and localizing eosinophils has led to speculation that it represents the next primary goal for therapeutic intervention in asthma, chronic obstructive pulmonary disease, and other eosinophilic diseases [9]. Many of these that are associated with tissue injuries related to eosinophilia are probably dependent on the same mechanisms playing roles in parasite protection. In cases of diseases like asthma, the cytotoxic potential of eosinophils that was originally developed to fight parasites can be directed against the host’s own tissue. Control over the use of this potential can therefore have positive effects in cases of such disorders. Moreover, studies indicate that the lifetime of tissues and their removal can be corrected by apoptosis and efferocytosis. The process of eosinophil apoptosis is delayed through IL-5, which is the typical cytokine responsible for eosinophil recruitment [10,11]. Eosinophilia has been shown to be a positive prognostic indicator for a number of human tumors. Many of these interesting possibilities for monitoring and manipulating the number of eosinophils may provide applicable means for anti-cancer therapy [12]. The possibilities of using of eosinophils for anti-tumor immunity is obvious and a new anti-tumor immunotherapy using a combination of T lymphocytes and eosinophils has been discussed [13].

## 3. Eosinophils as Antigen-Presenting Cells

Additional functions of eosinophils have been described on the basis of a finding that eosinophils can be induced to express major histocompatibility complex class II (MHC-II) and can work as antigen-presenting cells [14,15,16]. It was shown, however, that eosinophils create MHC-II 48 h after antigen stimulation [17]. Cytokines, including granulocyte macrophage colony-stimulating factor (GM-CSF), IL-3, IL-4, and interferon-gamma (IFN-γ) induce the expression of human leukocyte antigen—DR isotype (HLA-DR) on eosinophils [18].

Similarly, eosinophils die by programmed cell death (apoptosis), as do other cells (macrophage, dendritic cells etc.) [19,20], which the remainder of this review details.

## 4. Significance of Apoptosis of Eosinophil Granulocytes in Allergies and Asthma

Eosinophils contain a substantial number of histotoxic substances that contribute to the initiation and maintenance of allergic inflammatory reactions. Among these are cytotoxic protein granules, cytokines, and lipid mediators, each one of which plays a significant but different role. Although the purpose of eosinophils is adaptive immunity against parasitic diseases caused by helminths and flatworms, there is a commonly held opinion that products derived from eosinophils contribute significantly to allergic and asthmatic diseases [21,22]. The infiltration of bronchial mucosa by a large number of inflammatory cells is characteristic of the respiratory tract inflammation that is central to the pathogenesis of all forms of asthma. Although they have a minority position among the circulating leukocytes, they are distinctively dominant within this infiltrate. It was discovered that there is an increase of 50–100 times in the accumulation of eosinophils over neutrophils in the respiratory tracts of patients with asthma and chronic obstructive pulmonary disease [23,24]. Many studies exist showing a relationship between the severity of asthma and the number of eosinophils and their products in blood, in induced sputum, and in bronchial biopsy or lavage samples [25]. In addition, mediators derived from eosinophil granules participate significantly in damaging bronchial epithelial cells and leading to ciliary dysfunction and cell loss, both of which are regarded as central to the development of the bronchial hyperreactivity that is one of the main characteristics of asthma [26]. Even though the bronchial epithelium is commonly considered to be the target of cell damage by mediators derived from eosinophils, it has been observed that small human epithelial cells of the respiratory tract are also capable of engulfing apoptotic eosinophils. This is a specific process mediated by lectin and integrin that was extended to include the inflammatory cytokines IL-1α and tumor necrosis factor alpha TNFα [27,28]. Bronchial epithelial cells are capable of phagocytosing eosinophils but not neutrophils. These cells therefore play an important role in the elimination of apoptotic eosinophils from the airways in asthma [29]. This observation has significant consequences in relation to asthma in cleansing the lungs of eosinophils. Attention is being directed to understanding the factors participating in the removal of eosinophils, as eliminating the factors facilitating their removal by the apoptosis of eosinophils from the lungs and their subsequent engulfing by phagocytes is the therapeutic aim in asthma cases [30,31]. It has been shown that the absolute number of apoptotic eosinophils and macrophages in bronchial biopsies of asthmatic subjects is inversely correlated with their asthma symptoms as measured by the asthma severity score (ASS), which grades different forms of chronic asthma from very mild (score of 1) to very serious (score of 5) [32]. In other words, the milder the symptoms, the greater the percentage of eosinophil apoptosis in biopsy samples. The severity of asthma is correlated with an increased production of GM-CSF, which is itself related to the presence of non-atopic eosinophils. Moreover, GM-CSF inhibition of eosinophil apoptosis is an important aspect of eosinophil inflammation in asthma [19].

The dermal infiltration of eosinophils is characteristic of atopic dermatitis, lesions, and psoriasis in patients with this disease [33]. Eosinophils play an important role in atopic dermatitis. During atopic dermatitis, IL-4 and IL-5 are produced. IL-4 is then responsible for chemokine production and the initiation of the T helper 2 (Th2) cell response. IL-5 accelerates eosinophilopoiesis, and delays eosinophil apoptosis [34,35]. Cytokines such as GM-CSF, IL-3, and IL-5 are able to support the growth of the eosinophil population, but it is not clear if they mirror the enhancement of the de novo generation of eosinophils or the prevention of their apoptosis [36]. The three aforementioned cytokines can promote the survival of eosinophils by the nuclear factor kappa B (NF-kB) -induced upregulation of B-cell lymphoma-extra large (Bcl-xL), which can be blocked by specific inhibitors [37]. IL-5 is also involved in increasing Siglec-8-mediated cell death. Siglec-8 is cell surface receptor expressed on eosinophils and its ligation leads to cell death [38].

Nevertheless, it is more probable that the explanation lies in the very different methods employed by these studies in provoking allergic reactions. In the study of skin, there was one skin stimulus by an allergen, whereas in the study of nasal mucosa, two-day provocations were applied over the course of one week. When an aerosol allergen is inhaled by sensitive animals, the result is an inflammatory reaction and a permanent eosinophilia of the respiratory tract similar to the pathogenesis of human asthma. Eosinophils obtained through bronchoalveolar lavage of a sensitized mouse after stimulation by an aerosol allergen expressed Fas antigen, a cell surface receptor molecule, and were sensitive to apoptosis triggered by Fas. Even more important is the inhalation of anti-Fas monoclonal antibodies after the induction of lung eosinophilia as a result of the increase of peroxidase-positive macrophages in the bronchoalveolar lavage and a significant decrease in the number of eosinophils in the respiratory tract, which was related to the resolution of eosinophilic inflammation. It was also proved that the spontaneous recovery of a sensitive mouse from lung inflammation brought about by ovalbumin was related to the presence of eosinophil apoptosis in the bronchial subepithelium [39]. Anti-Fas monoclonal antibodies induce asthma-like inflammation in allergic airways. This seems to be due to the ability of anti-Fas to induce cytolysis of non-apoptotic eosinophils in airway tissues. Most apoptotic eosinophils progressed into secondary necrosis, which may exacerbate the inflammation [40].

## 5. Apoptotic Signals in Eosinophils

Apoptosis is a process of cell death. This process is characterized by the activation of intracellular caspases, the cleavage of DNA, the condensation and fragmentation of the nucleus, and plasma membrane blebbing. It leads to the phagocytosis of cell fragments without inducing an inflammatory response [20]. Apoptosis is important for the removal of eosinophils from the lungs [29]. When asthma occurs, the apoptosis of eosinophils is delayed [41]. Detailed knowledge of the mechanisms regulating this process creates the opportunity to establish projects focused on asthma treatment. There is extensive knowledge of cases where the accumulation of eosinophils occurs. Critical phases include increased eosinophil production in bone marrow, an increase in their release into the bloodstream, and their selective accumulation in the respiratory tract [42,43,44]. If eosinophils enter into tissue, there is no mechanism that would help them to migrate back after they fulfilled their role in an inflammatory allergic reaction. Their removal is dependent on their death, which is strictly controlled by the apoptosis mechanism, after which follows their recognition and phagocytosis by macrophages or resident cells. Hypotheses have been developed that the tissue burdened by large amounts of eosinophils in cases of allergic or asthmatic illnesses is related to the inhibition or dysfunction of apoptotic processes. Eosinophils are differentiated cells that die during apoptosis when cultivated in vitro, in which case they are quickly recognized as intact cells by autologous macrophages [45]. For two reasons, their phagocytosis is therefore essential for resolving inflammation and thus also asthma.

First, apoptosis prevents leakage of the histotoxic content of eosinophils, as is evident from cellular necrosis or cytolysis [40]. Even in a situation when eosinophils become apoptotic as the result of a failure or defect in the phagocytosis process, their removal would lead to release of their inflammatory content through secondary necrosis. Indirect evidence for this knowledge is provided by the observation that dermal eosinophils of patients with atopic dermatitis have not become apoptotic, but instead underwent cytolytic degeneration, in which cases have been shown to be associated with the deposition of products from eosinophilic granules in the dermis [46]. Second, the engulfing of apoptotic eosinophils induces the creation of anti-inflammatory cytokines and secretory profile mediators in macrophages; that is to say, IL-10, TGF-β, and prostaglandin E2, which is in direct contrast to the engulfing of necrotic eosinophils. This process is characterized by the presence of anti-inflammatory cytokines and mediators, which means releasing thromboxane B2 and GM-CSF [47]. Recognition and engulfing are dependent on phagocytic receptors distinguishing special changes in the membrane of apoptotic cells, commonly described as “eat me” signals [48]. At present, this condition is best characterized as the loss of phospholipid membrane asymmetry and of the phosphatidylserine contained within it that is usually limited to the inner cell membrane and during apoptosis is moved into the outer layer of the cell membrane [49]. Annexin V is a cellular protein (Ca^2+^ dependent) with high affinity for phosphatidylserine, and after marking by fluorescence stain, provides precise and early apoptotic signals in eosinophils [50,51]. It is clear that large quantities of non-professional phagocytes, including dendritic cells, smooth muscle cells, and lung fibroblast cells, are also capable of recognizing and engulfing apoptotic cells [52].

Necroptosis, which is a programmed form of necrosis requiring receptor interacting protein kinase-3 (RIPK3) and mixed lineage kinase like (MLKL) proteins, is an alternative pathway of cell death [53]. In eosinophils, necroptosis is modified by autophagy [54] because it is known that autophagy can promote RIPK1 degradation [55], and autophagy related protein 5 (ATG5) can activate necrosome formation in the autophagosome [56]. The regulation of eosinophil cytolysis and prevention of proinflammatory cell death in inflammatory responses based on eosinophils could be a new therapeutic possibility in bronchial asthma and atopic dermatitis [54,57].

## 6. Possibilities for Influencing Inflammation by Pharmacological Means

Many medicaments inhibit the production of eosinophils or of products derived from eosinophils. These include glucocorticoids, myelosuppressive drugs, leukotriene-synthesis inhibitors, leukotriene-receptor antagonists, tyrosine kinase inhibitors, IFN-α, IFN-β and anti-IL antibodies [58]. The etiology of a primary disease often determines the best therapeutic strategies. For example, patients with hypereosinophilic syndrome have a chromosome 4 deletion that results in the fusion of the *FIP1L1* and *PDGFRA* genes [59,60]. This gene fusion produces and activates tyrosine kinase, which is very sensitive to the inhibitor imatinib mesylate (Gleevec^TM^, Novartis Pharmaceuticals, Basel, Switzerland) that is used to treat some malignancies. Patients with eosinophilia along with *FIP1L1–PDGFRA*^+^ disease are treated in the first line by Gleevec^TM^ [61].

Moreover, there exists a number of other activated tyrosine kinases related to hypereosinophilic syndromes, including platelet-derived growth factor receptor-β (PDGFRB), Janus kinase-2, and fibroblast growth factor receptor 1 [62]. In the majority of individuals, glucocorticoids may provide the most effective means for reducing eosinophilia [63]. They can suppress the transcription of a number of anti-inflammatory mediator genes, including the genes for IL-3, IL-4, IL-5, GM-CSF, and various chemokines, including eotaxins. One of the main effects of glucocorticoids on cytokines activated by eosinophils is their possible participation in mRNA destabilization. This effect shortens the lifespan of cytokines such as eotaxins [64]. Apart from this, glucocorticoids inhibit the cytokine-dependent existence of eosinophils. Systemic or local treatment by glucocorticoids usually results in a significant decrease in eosinophils, but, in some patients, glucocorticoids are capable of maintaining eosinophilia despite the administration of high doses. The resistance mechanism of glucocorticoids is unclear, but a lowered level of glucocorticoid receptors and changes in the transcription factor activator protein-1 (AP-1) may play roles in the process [65]. A mechanism of resistance to glucocorticoid-induced apoptosis was introduced in activated eosinophils that is regulated by Pim-1-induced nuclear factor, interleukin 3 regulated (NFIL-3) and strengthened by the glucocorticoid receptor. By blocking Pim-1/NFIL-3 or eliminating the glucocorticoid receptor, glucocorticoid-mediated apoptosis in IL-5-activated eosinophils can occur. These findings support the development of new treatments for eosinophilic disorders resistant to steroid therapy [66].

In glucocorticoid-resistant patients, further treatment is sometimes needed, such as by means of myelosuppressive drugs (hydroxyurea, vincristine) or IFN-α [67]. IFN-α can be of particular benefit because it inhibits the degranulation of eosinophils and their effector function [68]. Particularly in patients with a myeloproliferative variant of hypereosinophilic syndrome, IFN-α therapy can often be left out. Cyclophilins (such as cyclosporine) have also been employed because they block the transcription of a number of cytokines (such as IL-5 and GM-CSF) [67]. It has been proven that lidocaine shortens the survival time of eosinophils. Its effects are similar to those of glucocorticoids, but it is not cytotoxic [69].

Clinical studies have shown that nebulized lidocaine is a safe and effective drug for asthma treatment [70]. Drugs that interfere with eosinophilic chemotactic signals include leukotriene antagonists and inhibitors. A 5-lipoxygenase inhibitor (such as zileuton) blocks the speed of a crucial step in leukotriene synthesis and inhibits the production of the eosinophilic chemoattractants LTB4 (leukotriene B4) and cysteinyl leukotrienes [71]. Cysteinyl leukotriene receptor antagonists block muscle contractions and increase vascular permeability intermediated by leukotrienes derived from leukocytes [72]. Some of the third-generation antihistamines inhibit the vacuolization [73] and accumulation [72] of eosinophils after an allergen stimulus and directly inhibit eosinophils in vitro [73,74]. Cromoglycate and nedocromil inhibit the effector functions of eosinophils such as antibody-dependent cellular toxicity [74]. The identification of molecules that specifically modify the function of eosinophils offers new treatment strategies. Substances that disrupt the adhesiveness of eosinophils to endothelium and interact with CD18/ intracellular adhesion molekule-1 (ICAM-1) [75] or very late antigen-4/vascular cell adhesion molecule-1 (VLA-4/VCAM-1) can be useful in this respect [76,77].

Antibodies against IL-5 have gone through clinical investigation [78,79]. Although their usefulness against asthma can be limited due to redundant pathways, anti-IL-5 is promising, especially for hypereosinophilic syndromes. Many inhibitors of the eotaxin/chemokine receptor type 3 (CCR3) pathway, including small molecule inhibitors of the CCR3 and human anti-eotaxin-1 antibody, are in development. Experiments with human anti-eotaxin-1 antibodies in patients with allergic rhinitis should prove the capability of this seemingly safe drug for reducing the level of nasal eosinophils and improving nasal patency [80]. The eosinophil surface molecule Siglec-8 offers therapeutic possibilities [81]. Siglec-8 is involved in the formation of a bond between sialic acid and lectin and contains immunoreceptor tyrosine based inhibitory motifs (ITIMs) that can provoke efficient eosinophilic apoptosis. Like CCR3 and chemoattractant receptor-homologous molecule expressed on Th_2_ lymphocytes (CR Th_2_). Therefore, these agents can be extraordinarily useful in treating allergic diseases and other autoimmune diseases [82].

There are intensively studied effects of various newer therapeutics, like mepolizumab or benralizumab, on eosinophil cell death and number. Mepolizumab (monoclonal antibody against IL-5) treatment decreases exacerbations and enhances Asthma Quality of Life Questionnaire (AQLQ) scores in patients with eosinophilic asthma. Eosinophils have an important role as effector cells in the pathophysiology of severe exacerbations of asthma [83]. Benralizumab blocks the IL-5 receptor; it is directed at the α subunit of the IL-5R. It is able to inhibit the differentiation of eosinophils in the bone marrow, as well as the eosinophilic infiltration of airways. Benralizumab decreases asthma exacerbations and decreases airflow limitations [84,85].

## 7. Process of Eosinophil Apoptosis

The apoptosis of lymphocytes is a process regulating the development of lymphocytes, as well as the functional potential of lymphocytes in diseases [86,87]. The same phenomenon occurs in granulocytes [88,89,90]. Errors in this process in eosinophils can contribute to eosinophilia being a factor in allergic diseases [91]. Eosinophils are inflammatory cells that primarily participate in the immune defense against parasites. In addition, they play an important role in the late stages of an inflammatory reaction and are prominent in many chronic inflammatory illnesses, particularly in allergic reactions such as bronchial asthma and atopic dermatitis [92]. Eosinophilia is directly related to clinical disease correlating with the activation of T lymphocytes and the production of specific cytokines [93]. Therefore, it is important to clarify the mechanisms that cause blood and tissue eosinophilia. In transgenic mice, eosinophilia occurred through an increased expression of IL-5 [94]. These observations were confirmed in some human inflammatory diseases. For instance, activated T lymphocytes were found in asthma, and it was found that these cells release soluble products that regulate eosinophilia in asthmatic patients [95]. The increased expression of several cytokines, including IL-3, IL-5, and GM-CSF, has been observed in cases of bronchial asthma [96].

IL-3, IL-5, and GM-CSF dramatically increase the lifespan of eosinophils by inhibiting their apoptotic cell death [97,98]. A hypothesis that the inhibition of eosinophil apoptosis through cytokines can have a key regulatory role in the development of eosinophilia exists [99].

It has been proven that tyrosine phosphorylation influences the inhibition and speeds up the apoptosis of eosinophils and neutrophils, even as it influences the survival of these cells [97]. In view of the fact that the activation of granulocyte apoptosis can be achieved directly or indirectly by phosphorylated proteins, the inhibition of eosinophil survival seems to be transcriptionally regulated. Regarding the fact that tyrosine phosphorylation plays the main role in transmembrane signal transfer over the majority of surface cell receptors, it should be possible to analyze those signal pathways regulating granulocyte apoptosis. As stated above, granulocyte apoptosis seems not to require de novo gene expression, which suggests that all death-related proteins are present in a mature cell and cannot be identified as inducible genes. On the other hand, cytokines that inhibit eosinophil apoptosis can induce “anti-death” genes, because both transcription and translation inhibitors annul their influence on the viability of cells [100,101]. Another candidate that can inhibit granulocyte apoptosis is a product of proto-oncogene *bcl-2*, which is known as an inhibitor of programmed cell death in many different types of cells [102]. Work in a transgene model has proven that *bcl-2* blocks the apoptosis of neutrophils, but not their phagocytosis [103]. There can be no doubt that the defective regulation of apoptosis is linked to eosinophilia [58]. We can assume that the inhibition of eosinophil apoptosis could contribute to eosinophilia, as observed in cases of asthma and other allergic disorders. Furthermore, the inhibition of granulocyte apoptosis can play an important role in other chronic inflammatory diseases. Therefore, it should be possible to develop therapeutic means to induce the apoptosis of specific eosinophils in patients with eosinophilic disorders. Apoptotic eosinophils could be then removed by macrophages without causing additional inflammation [104].

## 8. Research Perspectives in the Area of Eosinophil Apoptosis

Following on from the research [105] that the resolution of eosinophilic inflammation is probably caused by the induction of eosinophil apoptosis, these findings are not surprising as apoptosis is the most common form of physiological cell death. The relationship between eosinophilic inflammation and the apoptosis of eosinophils had already been described earlier in mice [106] and humans [107]. The importance of apoptosis for the removal of eosinophils in vivo is now very well known. Dysregulation of apoptosis can contribute to pathogenic processes. For instance, defective regulation of apoptosis can play a role in the etiology of cancer, acquired immune deficiency syndrome (AIDS), autoimmune diseases, and neurodegenerative disorders [102,103]. It is interesting that the process of eosinophil apoptosis is delayed in allergic inflammatory locations [81] and probably also in the blood of atopic patients [108]. Therefore, it seems that the delay in apoptosis contributes to the accumulation of these cells in chronic allergic inflammation, such as allergic rhinitis [58].

For instance, IL-4, an important cytokine in allergic reactions, induces VCAM-1 in microvascular endothelial cells. VCAM-1 binds to cells carrying the integrin VLA-4. The expression of VLA-4 in eosinophils, but not neutrophils, suggests that the VCAM-1/VLA-4 pathway contributes to a preference for the migration of eosinophils into allergic inflammatory locations [109,110]. Nevertheless, eosinophilia has also been observed under conditions where there was only very weak or no expression of IL-4 [111]. The ICAM-1/integrin CD11/CD18 pathway seems to be equally important for both eosinophils and neutrophils [112,113], which indicates that VCAM-1 induced by IL-4 cannot on its own explain the accumulation of eosinophils in allergic inflammation. Another possibility for achieving selectivity in the migration of circulating leukocytes into tissues is the differential expression of receptors for chemoattractants. For example, eotaxin is sent to selectively stimulated eosinophils [114]. The chemoattractants present in allergic tissues, such as platelet-activating factor (PAF) [115] and IL-8 affect both eosinophils and neutrophils [116,117].

It is improbable that the selective accumulation of eosinophils occurs exclusively on the level of chemotaxis. This view is strongly supported by observations in vivo [118]. It was proven that specific delays in eosinophil apoptosis play an important role in the development of tissue eosinophilia [119]. Activated T lymphocytes express a high level of IL-5, which functions as a survival factor due to its inhibition of the apoptosis of eosinophils, but not of neutrophils. The data discussed suggest that the accumulation of eosinophils in allergic tissues is achieved due to a complicated cascade of various processes. Many effects of eosinophil-derived cytokines related to the normal physiological functions of the body and to many diseases are known (Table 1) [120].

The expansion of eosinophils in tissues is regulated on the level of adhesion, chemotaxis, and apoptosis. Each step leads to a greater selectivity for eosinophils. How is the delay in eosinophil apoptosis in cases of allergic inflammatory reactions achieved? It is clear that the overproduction of survival factors, such as IL-5, is one important mechanism. Therefore, research is ongoing concerning the initiation of intracellular signal mechanisms through IL-5. These mechanisms mediate the anti-apoptosis of eosinophils [118,121]. In view of the fact that immature eosinophils strongly express Bcl-2 [122], it seems that Bcl-xl, but not Bcl-2, has anti-apoptotic effects in mature eosinophils [118].

Published articles suggest that eosinophil apoptosis is not regulated only in the presence or absence of survival factors. The death of eosinophils can be triggered also by surface death receptors. One of these death receptors expressed on eosinophils is CD95 (Fas/APO-1) [78,111,118,119]. CD95 ligand (CD95 L, FasL, APO-1 L) is highly expressed in activated T lymphocytes [119]. This means that the same cells that produce eosinophil survival factors also express at least one death factor for eosinophils. It is interesting that the activation of CD95 also occurs in the presence of eosinophil survival factors [78,121].

CD95L/CD95 molecular interactions can serve to restrict the expansion of eosinophils independently of the hematopoietic manifestation of eosinophils within an inflammatory location. Nevertheless, this process is much more complicated than it seems to be at first sight. For instance, nitric oxide, a secretory product released in increased amounts during a chronic eosinophilic inflammatory reaction, disrupts the CD95-induced signals of eosinophils. Studies have also unmistakably shown that eosinophils die by apoptosis in vivo [11,99,123,124].

**Figure 1:**
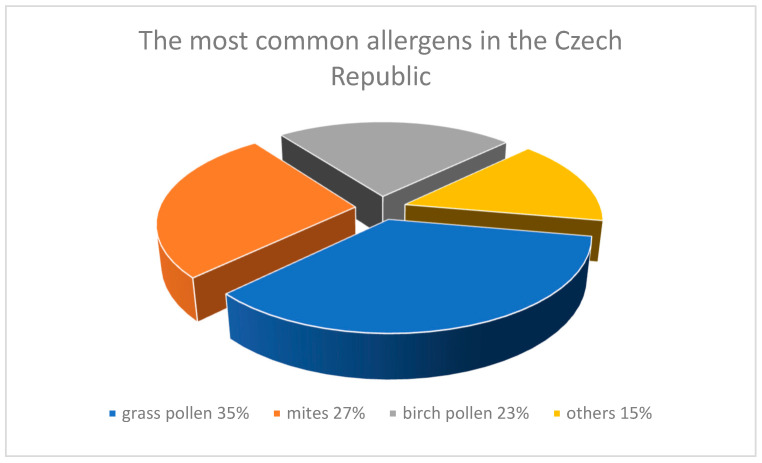
The most common allergens in the Czech Republic [125].

The studies of eosinophils in asthma and allergy are very important nowadays because of increasing numbers of cases of those diseases. For example, in the past 10 years, the number of people in the Czech Republic with allergies has doubled to surpass three million (Table 2, Figure 1) [125].

## 9. Conclusions

There is ever-increasing evidence that eosinophil apoptosis can by delayed in cases of allergic disorders. There are at least two mechanisms responsible for this: the increased expression of eosinophil survival factors and the disruption of death signals. The identification of molecules participating in anti-apoptotic pathways in eosinophils offer hope for the development of new drugs to reduce the number of eosinophils and, accordingly, eosinophilic inflammation in cases of allergic diseases.

## Figures and Tables

**Table 1:** Effects of cytokines produced by eosinophils [120].

Functions	Cytokines
Developmental functions	TGF-beta, IL-4
Cell–cell interactions	Interleukins (1, 3, 4, 5, 6, 8, 9, 12, 13), IFN-γ, TGF-β, TNF-α, NGF, SCF, GM-CSF, CCL5, CCL11
Metabolic homeostasis	IL-4, IL-13
Immune polarization	Interleukins (4,5,10, 12, 13, 18, 25, 33), IFN-γ, TGF-β, CXCL9, CXCL10, CCL17, CCL22
B cell maintenance	Interleukins (4, 5, 6, 10), TNF, APRIL
Tissue repair/remodeling	Interleukins (1β, 6, 13), PDGF, SCF, TGF-α, TGF-β, VEGF, CXCL1, CXCL10, CXCL12, CCL2, CCL3, CCL11

**Table 2:** Allergies in the Czech Republic [125].

Types of Allergies	Number of Czech People
Allergic rhinitis	2.5 million
Asthma	1.0 million
Atopic eczema	0.8 million
Food allergy	0.4 million
